# Structural and Compositional Effects of Essential Oils on Silica-Rich Gel Materials for Novel Dental Applications

**DOI:** 10.3390/polym18162000

**Published:** 2026-08-17

**Authors:** Hanne Bulut, Osman Arslan, Chi Ching Lee

**Affiliations:** 1 Hem Group, Zaim Technopark, 34303 Istanbul, Türkiye; hannebulut00@gmail.com; 2 Food Engineering Department, Sabahattin Zaim University, Küçükçekmece, 34303 Istanbul, Türkiye; chi.lee@izu.edu.tr

**Keywords:** essential oils, gel materials, dental applications, cytotoxicity

## Abstract

Particle-containing gels were functionalized with clove, peppermint, tea tree, sage, coconut, lemon, and eucalyptus oils to obtain a novel material for dental applications with improved cell interactions and antibacterial applications. Dried samples, SEM imaging, and elemental mapping revealed a homogeneous distribution of inorganic and organic components throughout the gel matrix. EDX and XPS investigations revealed changes in elemental composition and surface chemistry following the addition of essential oil. Also, FTIR spectroscopy revealed characteristic interactions between the silica-rich network and bioactive oil constituents through slight modifications. In contrast, UV–Vis and PL investigations showed differences in the optical behavior of the formulations. TG-DTA measurements with decomposition analysis revealed a noticeable increase in essential oils, which broadly influenced the chemical composition. Biological evaluation using L929 fibroblasts demonstrated >73% cell viability after 72 h in essential oil-containing structures, confirming proper cytocompatibility. The compatibility between the silica-based formulation and essential oils resulted in better cellular effects without compromising compatibility. Therefore, the promising potential of these multifunctional formulations for advanced oral care applications has been experimentally demonstrated and reported.

## 1. Introduction

Gels and gel-like materials are three-dimensional networks that can modulate and entrap large amounts of liquid while maintaining structural integrity through physical or chemical crosslinking [1,2,3]. Their controllable chemical and physical properties can enable widespread applications in the pharmaceutical, biomedical, cosmetic, and oral healthcare fields [3,4,5]. Among these systems, silica-rich gel matrices are widely used in modern dentifrices for their excellent biocompatibility, chemical stability, polishing efficiency, and capacity to accommodate biologically active compounds [6,7]. These characteristics make silica-based gels attractive as multifunctional platforms for incorporating antimicrobial and therapeutic agents to improve oral health [8]. Essential oils have attracted considerable attention as natural multifunctional additives owing to their antimicrobial, antioxidant, anti-inflammatory, analgesic, and antifungal activities [9,10,11,12,13,14,15,16,17,18,19]. Their biological effects generally arise from multiple mechanisms due to the disruption of microbial membranes, inhibition of enzymatic pathways, suppression of biofilm formation, and modulation of oxidative stress [20,21]. Tea tree oil including terpinen-4-ol, peppermint oil including menthol, sage oil, lemon oil (limonene), coconut oil (lauric acid), and clove oil (eugenol) showed antibacterial and anti-inflammatory activities relevant to oral healthcare, making them promising natural alternatives to synthetic antimicrobial agents [22,23,24].

**Figure 1:**
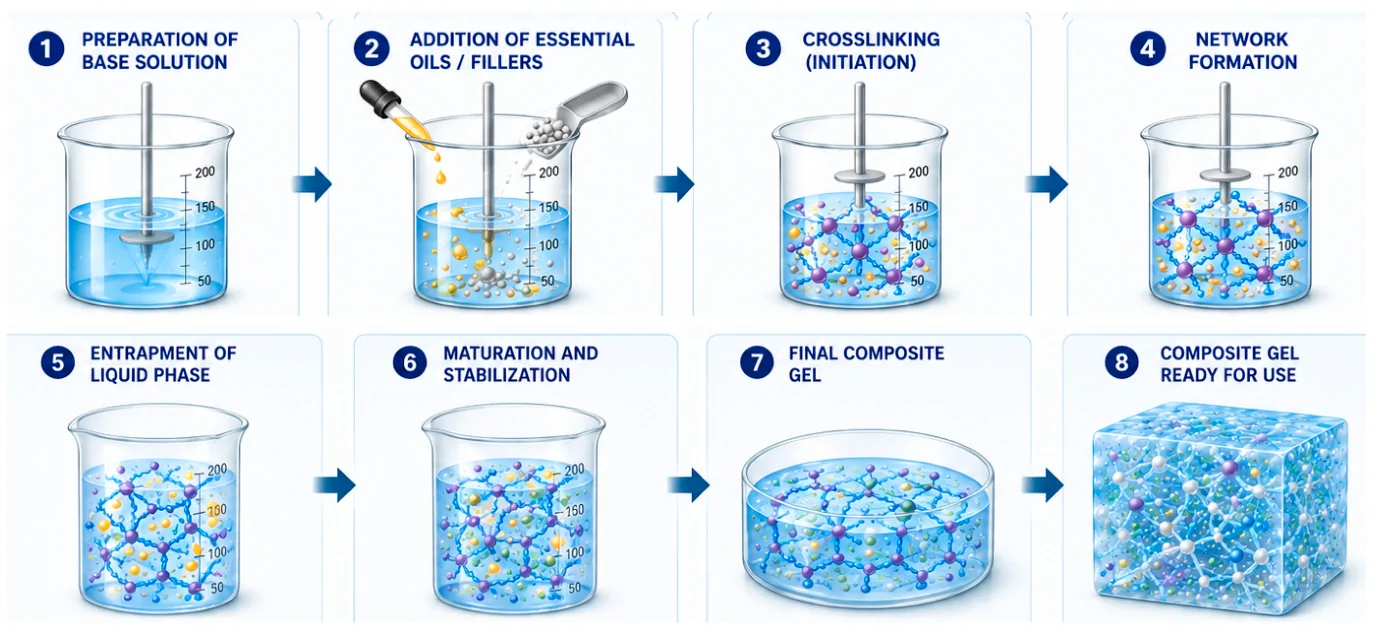
Addition of the special essential oils into the designed composition for the preparation of novel composite gel materials.

The oral medium is continuously exposed to microbial biofilms, mechanical abrasion, and fluctuating pH, which is important for the use of antimicrobial dental materials and is highly desirable [25,26,27]. Silica-rich gel formulations containing ZnO provide an ideal platform for incorporating natural essential oils, combining structural stability with enhanced antibacterial performance and favorable biocompatibility [28,29]. Therefore, this study investigates the physicochemical, structural, thermal, optical, cytocompatibility, and antibacterial properties of silica-rich, ZnO-containing dental gels functionalized with clove, peppermint, tea tree, sage, eucalyptus, lemon, and coconut oils for advanced oral care applications, and Figure 1 clearly represents the steps of the new preparation [30,31].

Comprehensive studies investigating the effects of combining various natural essential oils with ZnO in a silica-rich gel system and systematically evaluating their physical/chemical properties through the use of complementary structural, morphological, optical, thermal and surface analytical techniques are still lacking. The current study developed a silica-rich gel formulation that included ZnO and a mixture of seven natural essential oils (clove, peppermint, tea tree, eucalyptus, sage, lemon, and coconut) and compared them. The main purpose of this study was to determine how the addition of these bioactive ingredients affected the structure, surface chemistry, optical properties, and thermal stability of the gel while maintaining the structural integrity of the silica-rich matrix. To the best of our knowledge, such a comprehensive physicochemical characterization of a multi-essential-oil-enriched silica-rich ZnO gel intended for dental applications has not been previously reported.

## 2. Materials and Methods

### 2.1. Materials

Basic gel materials containing hydrated silica, sorbitol, glycerin, xanthan gum, lauryl glucoside, and cocamidopropyl betaine were obtained from Merck and Sigma Aldrich. Modifications were made to this formulation by adding eucalyptus globulus leaf oil, citrus lemon peel oil, seatons coconut oil, eugenia caryophyllus leaf oil, mentha piperita oil, salvia triloba leaf oil, and melaleuca alternifolia leaf oil which were obtained from Merck and Sigma Aldrich. Pure water was used for dispersion purposes.

### 2.2. Preparation of the Gel Structures

Glycerin and xanthan gum are mixed and stirred until a homogeneous mixture is obtained. Then sorbitol is added and heating is applied together with water addition. The silica is transferred to the system together with ZnO and water is added to the mixture. After the addition of lauryl glucoside and cocamidopropyl betaine, controlled cooling is applied to the mixture. Aloe vera extract and menthol are added for the final composition. This composition was named S1, and after the addition of essential oils, such as aloe vera extract, fragrance, menthol, coconut oil, citrus oil, eucalyptus oil, peppermint oil, clove oil, sage oil, and tea tree oil, another composition was obtained, which was named S2.

### 2.3. Characterization

An X-ray diffractometer (XRD) (Malvern PANalytical X’Pert PRO) was used to determine the X-ray diffraction pattern of the dried gel materials with CuK_α_ radiation in the 2θ = 5–90 range. The elemental composition investigations (EDX mapping) and atomic mapping of the dried gel materials were performed together with morphology and diameter analyses with a scanning electron microscope (SEM) (Thermo Scientifıc Apreo 2 S LoVac). In order to avoid the electron charging effect, the surface of the samples was coated with 5 nm Au/Pd prior to SEM imaging. X-ray photoelectron spectroscopy (XPS-Thermo Scientific K-Alpha) spectra were obtained by a flood gun charge neutralizer system equipped with a monochromated Al Ka X-ray source (hν = 1486.6 eV) from a 400 mm spot size on the nanofibrous web. Wide energy survey scans were recorded in the 0–1360 eV binding energy range, at a detector pass energy of 200 eV, and with an energy step size of 1 eV. High-resolution spectra were obtained at a pass energy of 50 eV with energy steps of 0.1 eV for each atom. The optical behavior of the structures was observed by UV-vis spectrophotometry (Optima SP 3000) and a fluorescence spectrophotometer (Horiba, Fluoromax 4). Surface features, interactions, and chemical reactivity, together with functional groups, were detected by FT-IR spectroscopy scanning between 400 cm^−1^ and 4000 cm^−1^ by Shimadzu-IR Tracer-100 with ATR module. The obtained powders’ thermal properties were compared between room temperature and 900 °C with Seiko EXSTAR SII 6300 TGA-DTA. A nitrogen atmosphere was used as the decomposition environment.

### 2.4. Drying Procedure for the Gel Materials

**Figure 2:**
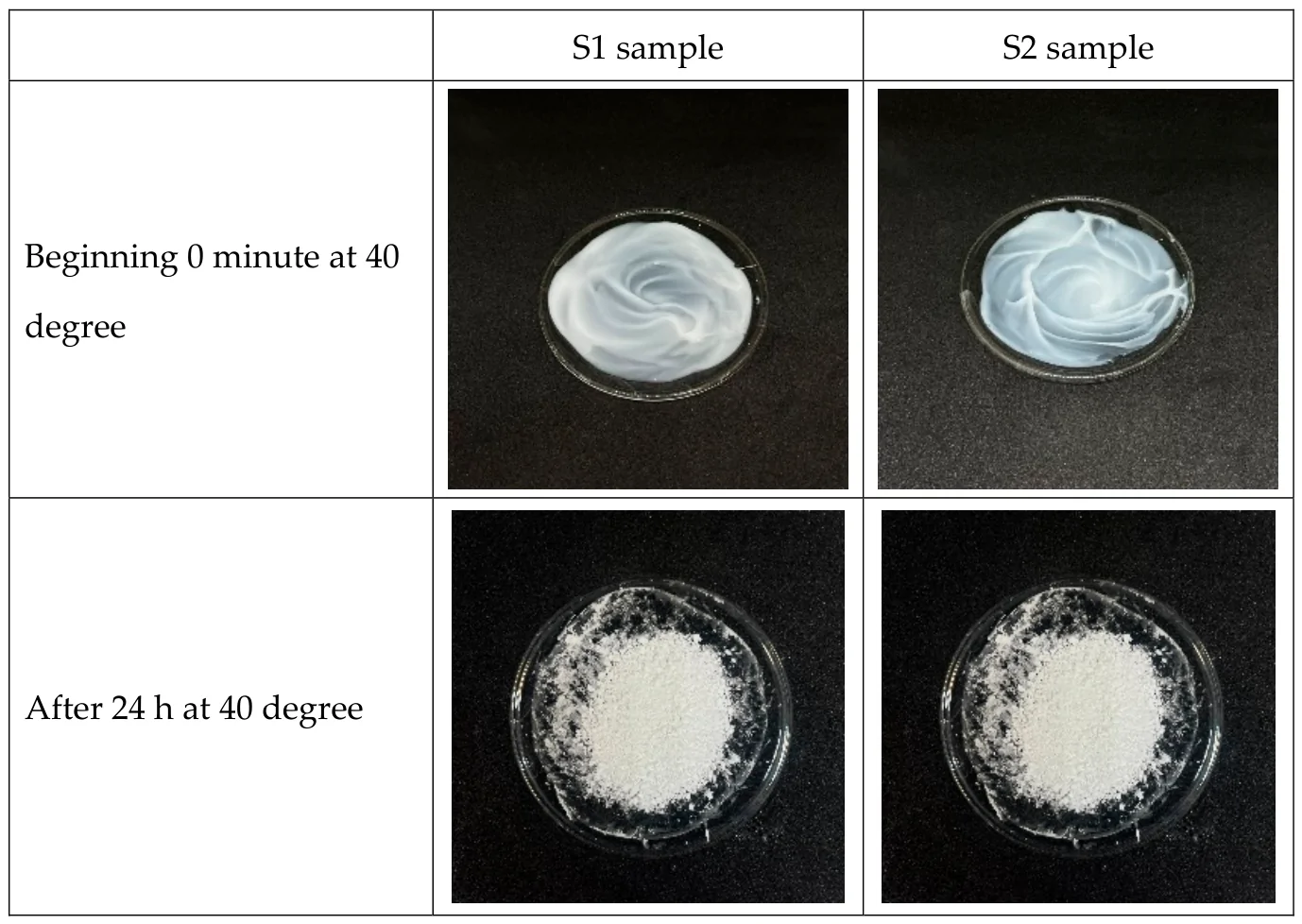
Gel and dried powder images of the S1 and S2 formulations.

After preparation and homogenization, gel formulations of S1 and S2 (Figure 2) were allowed to equilibrate at room temperature (25 ± 2 °C) for 12 h to ensure uniform distribution of the volatile components throughout the gel matrix. Drying was subsequently performed in an oven under mild conditions to minimize the evaporation and degradation of the essential oil constituents. Approximately 5–10 g of each formulation was uniformly spread as a thin layer (2–3 mm thickness) in glass Petri dishes and transferred to the oven maintained at 40 °C for 24 h.

These drying conditions were selected to remove residual moisture while preserving the volatile phytochemicals incorporated into the gel. After drying (Figure 2), the samples were cooled to room temperature in a desiccator containing silica gel to prevent moisture uptake from the atmosphere. The dried gels were then stored in airtight glass containers at room temperature until further physicochemical characterization. This controlled drying protocol minimized the loss of volatile compounds while maintaining the structural integrity and chemical stability of the composite gel formulations. Since SEM analysis requires completely dry and vacuum-compatible specimens, the essential oil-containing gel formulations underwent a modified drying procedure prior to morphological examination. After preparation, a small amount of each gel sample was spread as a thin layer on clean glass substrates and pre-equilibrated at room temperature to allow partial moisture removal without disrupting the gel structure. Finally, the dried specimens were stored in a desiccator until analysis and coated with a thin conductive layer prior to SEM imaging. This modified sample preparation protocol enabled the evaluation of the gel morphology while reducing structural collapse and moisture-related artifacts. Also, gel and dried samples were subjected to thermal analysis to examine the volatile content.

### 2.5. Cytotoxicity Tests

The L929 cell line that was collected from Istanbul Sabahattin Zaim University was used. L929 was incubated at 37 °C, 5% CO_2,_ and 95% humidity conditions and supplied with DMEM low glucose (Gibco LOT:2242412) with 10% FBS and 1% penicillin–streptomycin (Sartorius LOT:223603612) media. Cells were grown up to 80% competency of the T75 flask surface. Then, the cells were trypsinized, and the effect of trypsin was neutralized by the addition of the corresponding media into the cell culture environment. After that, cells were collected by centrifugation at 1300 rpm at room temperature. The supernatant was discarded, and cells were used for further experiments. Cells were seeded in 96-well plates (Wuxi, China, NEST Biotechnology LOT: 04522BL01) with 104 cells per well. After waiting for 24 h, cells were treated with different concentrations of dried toothpaste samples. Before treatment, dried toothpaste was exposed to UV light for 30 min for sterilization. Then, it was dispersed in DMEM low glucose (Gibco LOT:2242412) with 1% penicillin–streptomycin (Sartorius LOT:223603612) media in a 200 mg/mL concentration. After that, it was incubated at 37 °C, 5% CO_2_, and 95% humidity conditions for 24 h. Next, the liquid phase was transferred into a new tube and it was used to make 100, 50, 25, 12.5, and 6.25 mg/mL concentrations that cells were exposed to for 24 h. Then, 10 µL MTT solution (5 mg/mL concentration) was added to the cells and incubated for 4 h. After incubation, the media with the MTT solution was discarded, and 100 µL DMSO was added to the cells to dissolve the formazan crystals. The absorbance was read at 590 nm in a spectrophotometer (Agilent Technologies, Biotek 800 TS Microplate reader).

## 3. Results and Discussion

### 3.1. Crystallinity and Thermal Properties

**Figure 3:**
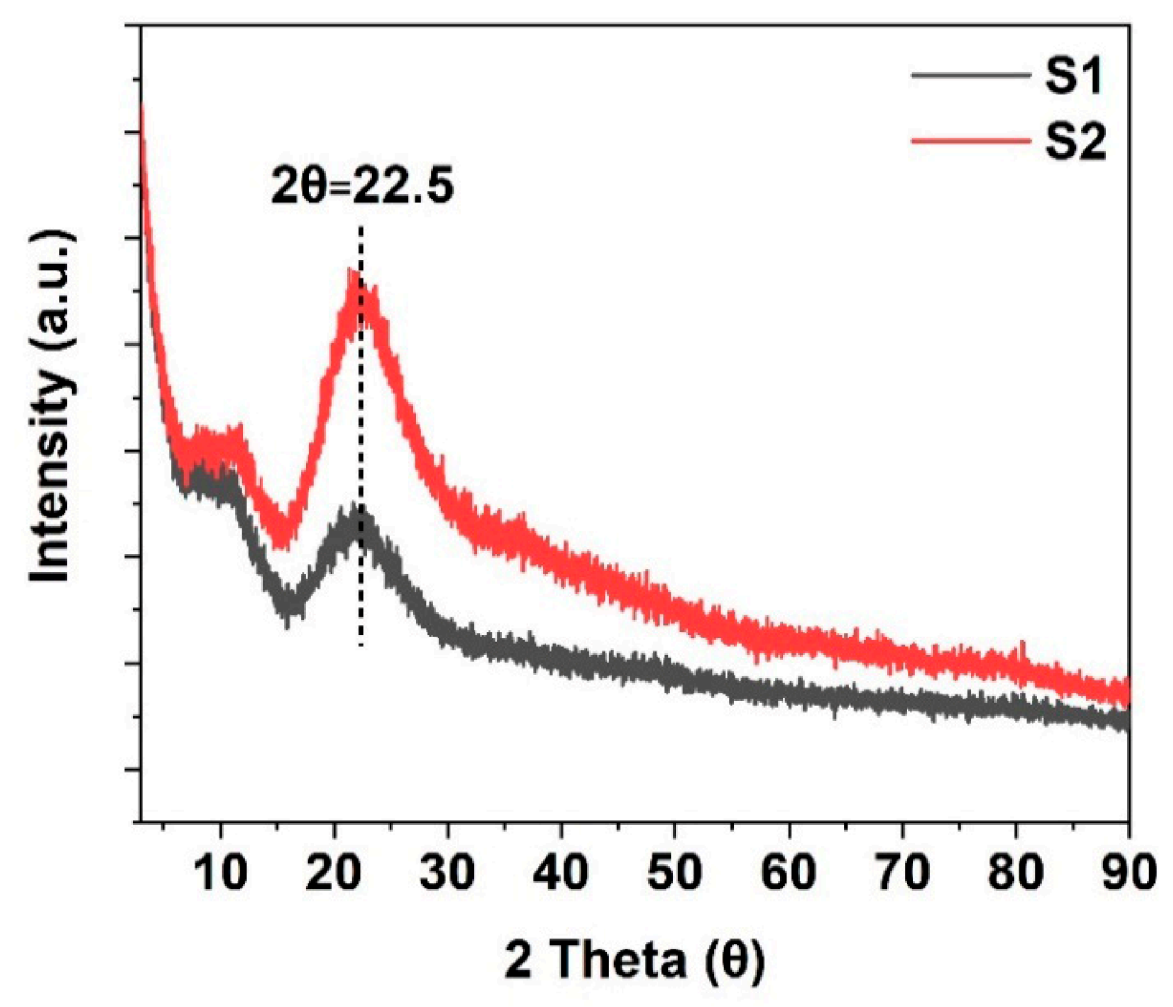
XRD patterns for the S1 and S2 gel formulations.

X-ray diffraction (XRD) investigation was conducted to evaluate the crystalline behavior of the silica-rich gel formulations. As expected, both the S1 and essential oil-containing (S2) formulations exhibited broad diffraction peaks centered at 22.5° (2θ), confirming the predominantly amorphous nature of the silica matrix (Figure 3) [32,33]. The observed small peak shift and different diffraction intensity for S2 essentially mean that enhanced short-range structural organization is possible due to intermolecular interactions between the essential oil constituents and the silica network. No additional crystalline diffractions were observed, indicating that the addition of essential oil did not induce new crystalline phases or alter the structural integrity of the gel matrix. It is also clear that additional diffraction peaks for the ZnO structure were not observed. Diffraction results revealed that the essential oils did not cause significant changes in the silica skeleton or the composite gel formulations. An XRD peak was observed at around 10° (2θ), and because it has low intensity, this peak can be attributed to short-range organization. This peak may also originate from local packing within the hydrated silica structure and interactions among silanol groups and residual organic constituents. This low-angle shoulder clearly suggests nanoscale structural heterogeneity or partially ordered organic–inorganic amorphous domains within the gel matrix.

**Figure 4:**
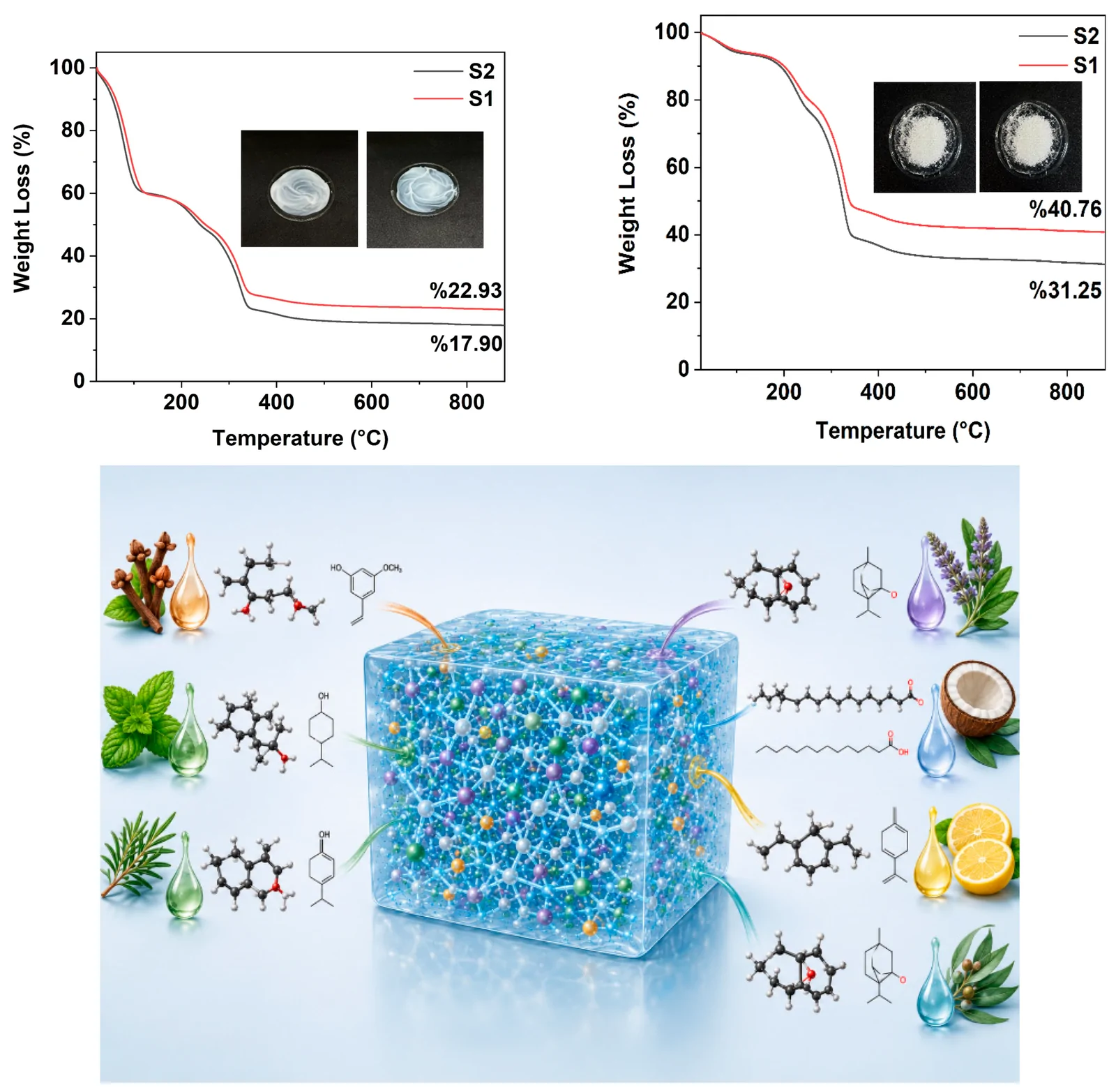
TGA profiles of the S1 and S2 gel formulations (**upper left**) and TGA profiles of the dried powder formulations (**upper right**), table of thermal losses for different regions, and essential oil addition into the designed gel formulation.

Thermal decomposition patterns of the gel (S1) and the essential oil-containing gel (S2) revealed the same multistep thermal degradation profiles, characteristic of silica-based composite gels (Figure 4). The first weight loss below 120 °C can be attributed to moisture removal (possibly water and other volatile constituents), followed by the gradual decomposition of bound water and low-molecular-weight organic constituents between 120 and 280 °C. The slightly earlier degradation of S2 reflects the presence of essential oil components, as they are generally highly volatile [34,35]. Organic decomposition primarily begins between 280 and 360 °C and is possibly responsible for the degradation of the organic gel network composed of polyols and other organic components. At 900 °C, S1 retained a higher residual mass (22.93 wt.%) than S2 (17.90 wt.%), owing to the additional volatile organic fraction introduced by the essential oils. From Table 1, we can understand that the most notable difference is that the essential oil addition affected the residual amount when subjected to the same temperature limits when compared to other samples. It seems that the most mass loss was obtained for the S2 sample without drying.

Still, S1 and S2 revealed noticeable degradation pathways in the absence of additional thermal effects, and the addition of essential oil preserved the thermal durability and structural integrity of the silica-rich skeletons. To further evaluate the intrinsic thermal behavior of the inorganic-rich matrix, the dried powder forms of both formulations were subsequently analyzed by TGA. The TGA curves of the completely dried gel (S1) and the essential oil-containing gel (S2) demonstrate the intrinsic thermal stability of the composite formulations (Figure 4) after effective moisture removal. When compared with the dried gel samples, the observed weight loss below 120 °C simply confirms that residual water was removed prior to analysis, allowing the observed thermal events to be attributed to degradation of the gel matrix and added organic constituents. Both S1 and S2 formulations exhibit a similar multistep degradation profile, with the principal decomposition occurring between approximately 250 and 360 °C, corresponding to the breakdown of the polymeric gel network and bioactive components. In fact, the decomposition profile is almost analogous to the gel structure. The essential oil-containing formulation (S2) exhibits a slightly greater mass loss in this region, reflecting the thermal decomposition of additional volatile phytochemicals. This situation is expected, since gel has many hydrogen-bonding and polar interactions between the essential oils and the matrix formulation. At 900 °C, the final mass of S1 (40.76 wt.%) is higher than that of S2 (31.25 wt.%), showing that the essential oil addition (Figure 4) increases the proportion of thermally degradable organic matter while reducing the final inorganic residue. Still, no other degradation was observed, and the similar decomposition pathways confirm that incorporating essential oils does not alter the fundamental thermal degradation mechanism of the silica-based composite gel, but only modifies its quantitative thermal behavior. Consequently, TGA curves were analyzed using four thermal regions (to evaluate the relative contributions of the degradation processes). Mass loss at temperatures below 100 °C was primarily due to physically adsorbed water. Degradation in the range of 100–225 °C is thought to occur as a result of loss of bound water and low-molecular-weight volatiles. Decomposition of the organic matrix of the gel and of the volatile organic components derived from the essential oil is thought to be responsible for the largest degradation event that takes place in the temperature range of 225–360 °C. At temperatures above 360 °C, there was a steady degradation of residual organic matter leading to the formation of carbonaceous residue. The remaining mass after thermal treatment was primarily composed of the thermally stable inorganic component (silica/zinc oxide). Corresponding mass losses for gel and dried forms of S1 and S2 are listed in Table 1 for four regional temperatures.

### 3.2. Optical Features of the Gel Materials

**Figure 5:**
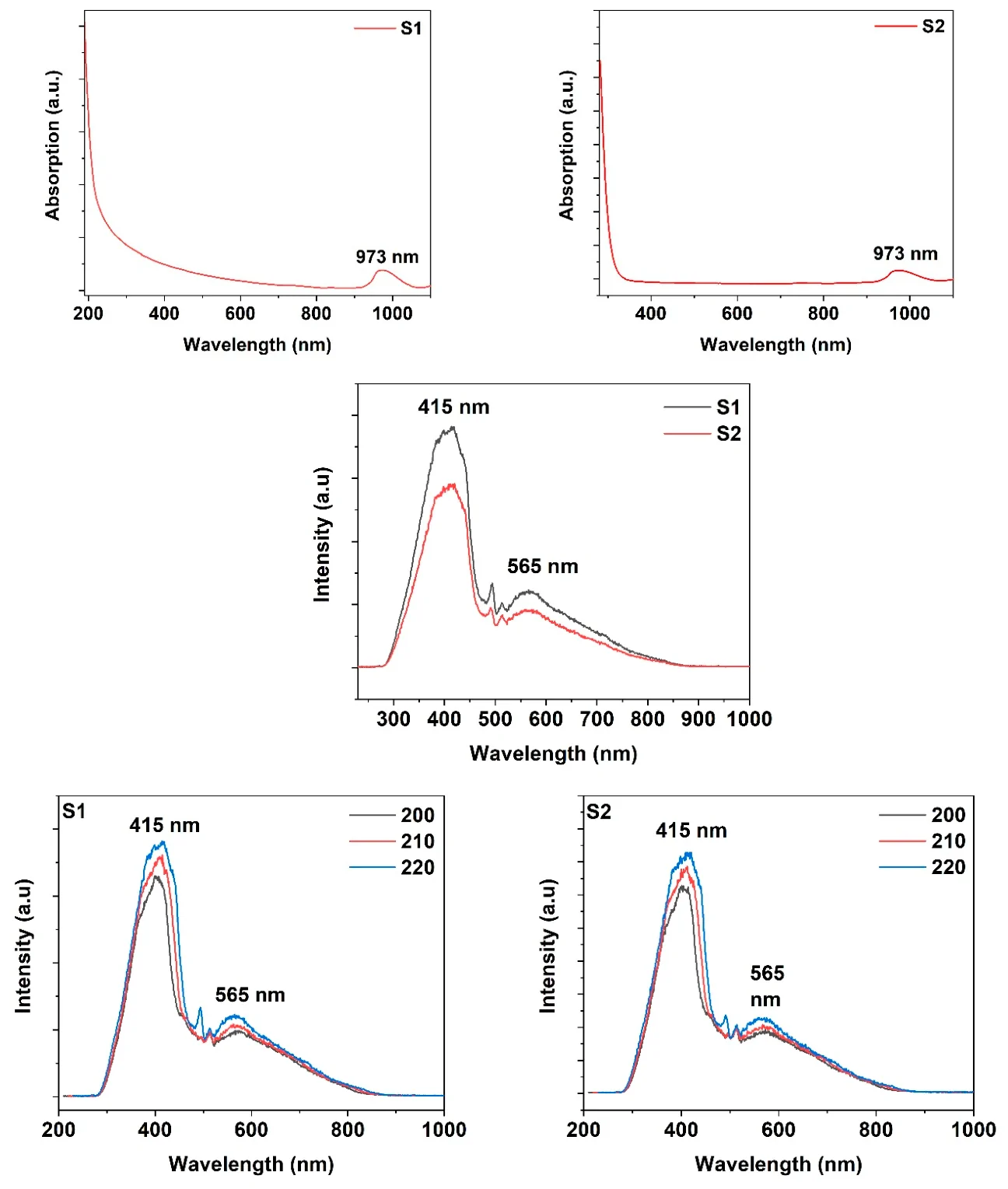
Uv-Vis investigation of the S1 and S2 formulations (**up**); (**middle**) overlapped photoluminescence investigation of the S1 and S2 formulations; (**down**) separate photoluminescence investigation of S1 and S2.

The UV–Vis graph of S1 and the essential oil-added formulation (S2) (Figure 5) shows generally similar spectral characteristics throughout the measured wavelength range, indicating that the incorporation of essential oils has only a small variation in the optical behavior of the silica-based gel matrix. Both samples display intense absorption in the ultraviolet region (200–275 nm), followed by a progressive decrease toward the visible region. In particular, the 200–300 nm region is characterized by a steep decline in absorbance, which is associated with high-energy electronic transitions, including π-π* and n-π* transitions originating from oxygen-containing functional groups and aromatic or unsaturated phytochemical constituents present in the formulation [36,37]. Especially for S2, the incorporation of double-bond- and carbonyl-containing essential oils may cause this shift in optical properties. The more pronounced decrease observed for S2 in this region suggests that incorporating essential oils modifies the local electronic environment of the gel matrix via intermolecular interactions rather than by forming new chromophoric species. These interactions may involve hydrogen bonding between silanol groups and oxygenated compounds such as eugenol, menthol, terpinen-4-ol, limonene derivatives, and 1,8-cineole, resulting in slight changes in the UV absorption profile while preserving the overall spectral characteristics. Since silica surface structures interact with almost all added species, an expected alteration is possible. No other absorption peaks were detected, supporting the conclusion that the essential oils are homogeneously attached at the molecular level within the composite gel, without inducing significant structural modifications or aggregation. The absorption band observed at approximately 973 nm in both S1 and S2 is mainly attributed to near-infrared overtone absorption of O–H stretching vibrations associated with physically adsorbed water and surface silanol groups (Si–OH) in the hydrated silica-rich gel matrix [38]. Since this 973 nm peak is present in both formulations, we conclude that it is a feature of the hydrated silica-based system and not an absorption peak attributable to essential oil incorporation. The comparable positions of the band in S1 and S2 indicate that the fundamental hydration environment and the gel’s silanol-rich network are preserved after the addition of essential oil. The comparable position of this band demonstrates that the molecular environment of the inorganic matrix remains essentially unchanged after essential oil incorporation. However, a peak observed for S2 throughout the visible and near-infrared regions supports improved optical homogeneity and reduced light scattering, which may result from a more uniform distribution of organic molecules within the silica skeleton. In fact, SEM observations strongly support this phenomenon. The photoluminescence graphs (Figure 5) of S1 and S2 show two noticeable emissions upon excitation at 210 nm, with a strong blue emission centered at approximately 415 nm and a broader green–yellow emission band centered at approximately 565 nm. The intense 415 nm emission observed in both formulations indicates the presence of optically active centers within the silica-based composite gel, which may be associated with surface defect states, oxygen-related centers, ZnO-related emission sites, or organic–inorganic interfacial states. The broad nature of this emission suggests that the luminescence originates from multiple overlapping radiative recombination pathways rather than from a single well-defined electronic transition. The persistence of this peak in both S1 and S2 confirms that the fundamental emissive structure of the composite matrix is preserved after essential oil incorporation, and that it did not alter the main luminescence features. Compared with S1, the essential oil-containing formulation S2 shows a clear decrease in emission intensity. When deeply analyzed, the PL spectra obtained by three different excitations (200 nm, 210 nm, 220 nm) (Figure 5) for S1 and S2 show that intensity reduction suggests that essential oil molecules interact with the emissive centers of the composite gel and partially suppress radiative recombination, most likely through surface adsorption, hydrogen bonding, polarity changes, or non-radiative energy transfer processes. There is no significant change in wavelength upon the addition of essential oil. Also, the addition of essential oils does not form new emissive states or significantly change the matrix’s electronic structure. Instead, the essential oils mainly modulate the emission efficiency of the existing luminescent centers [37,39]. These results support the successful incorporation of essential oils into the gel network while maintaining the gel system’s characteristic photoluminescent behavior.

### 3.3. Atomic and Structural Properties with Detailed SEM Investigation

**Figure 6:**
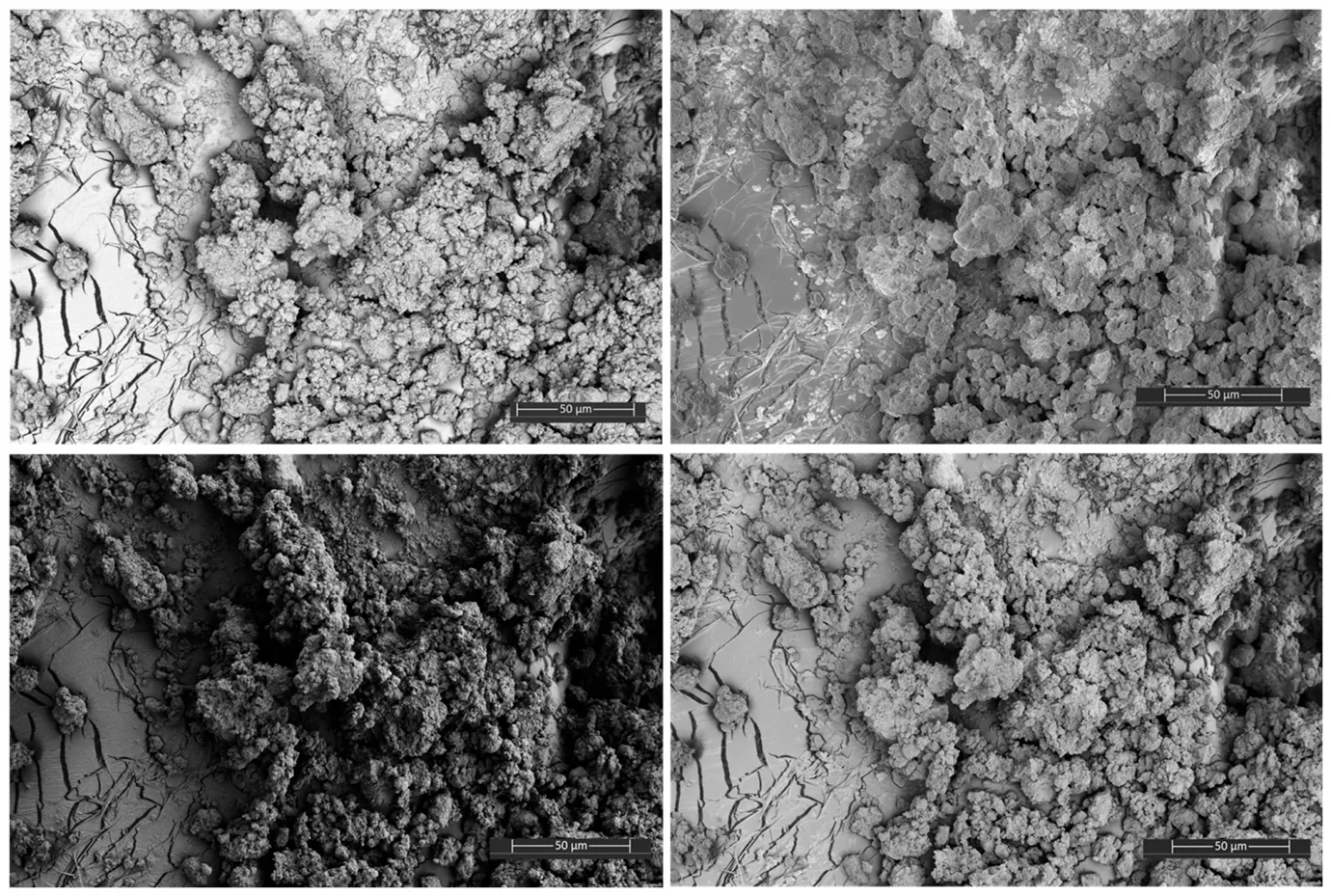
T1, T2, ETD, and Mix SEM images of S1 with ×1500 magnification.

**Figure 7:**
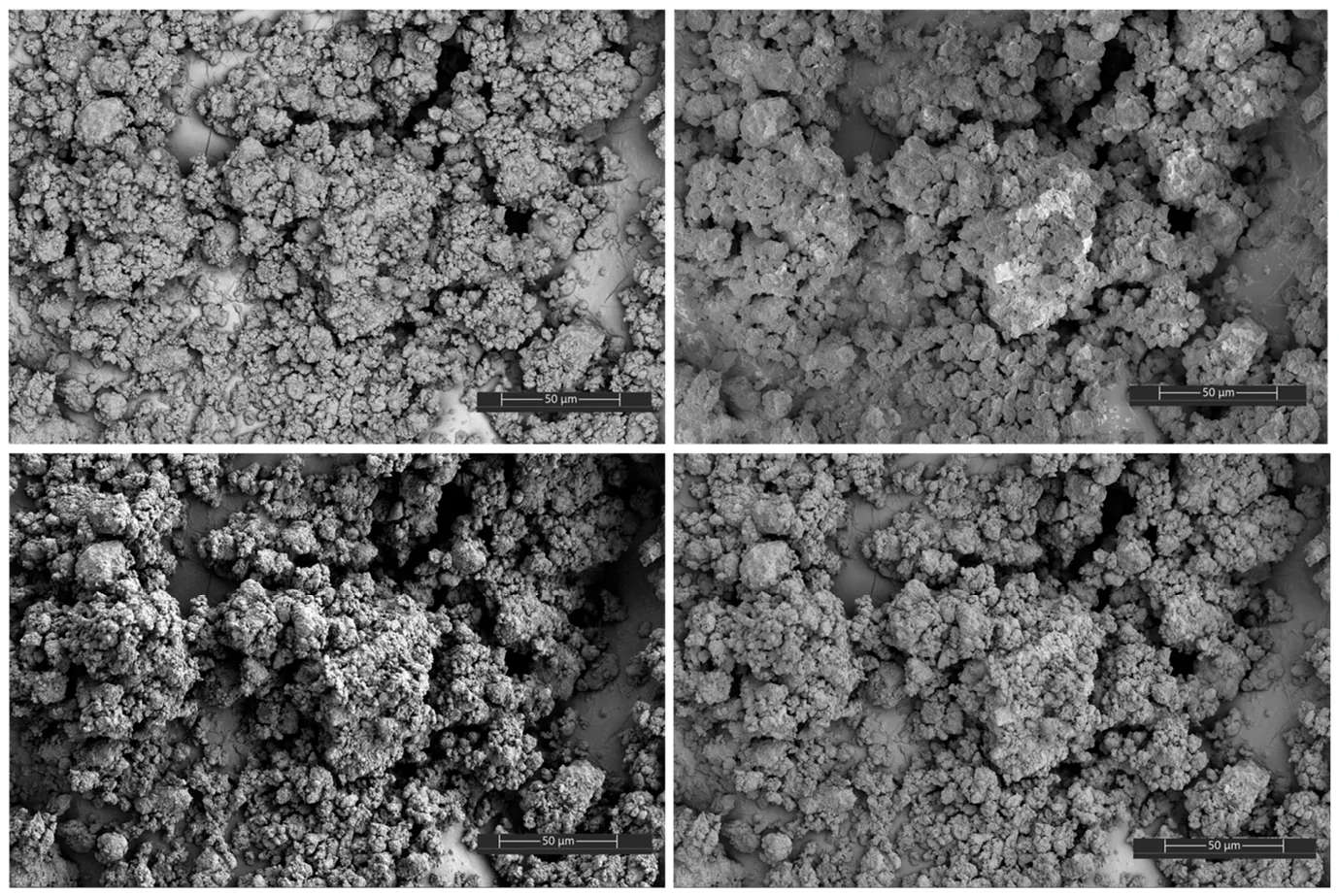
T1, T2, ETD, and Mix SEM images of S2 with ×1500 magnification.

**Figure 8:**
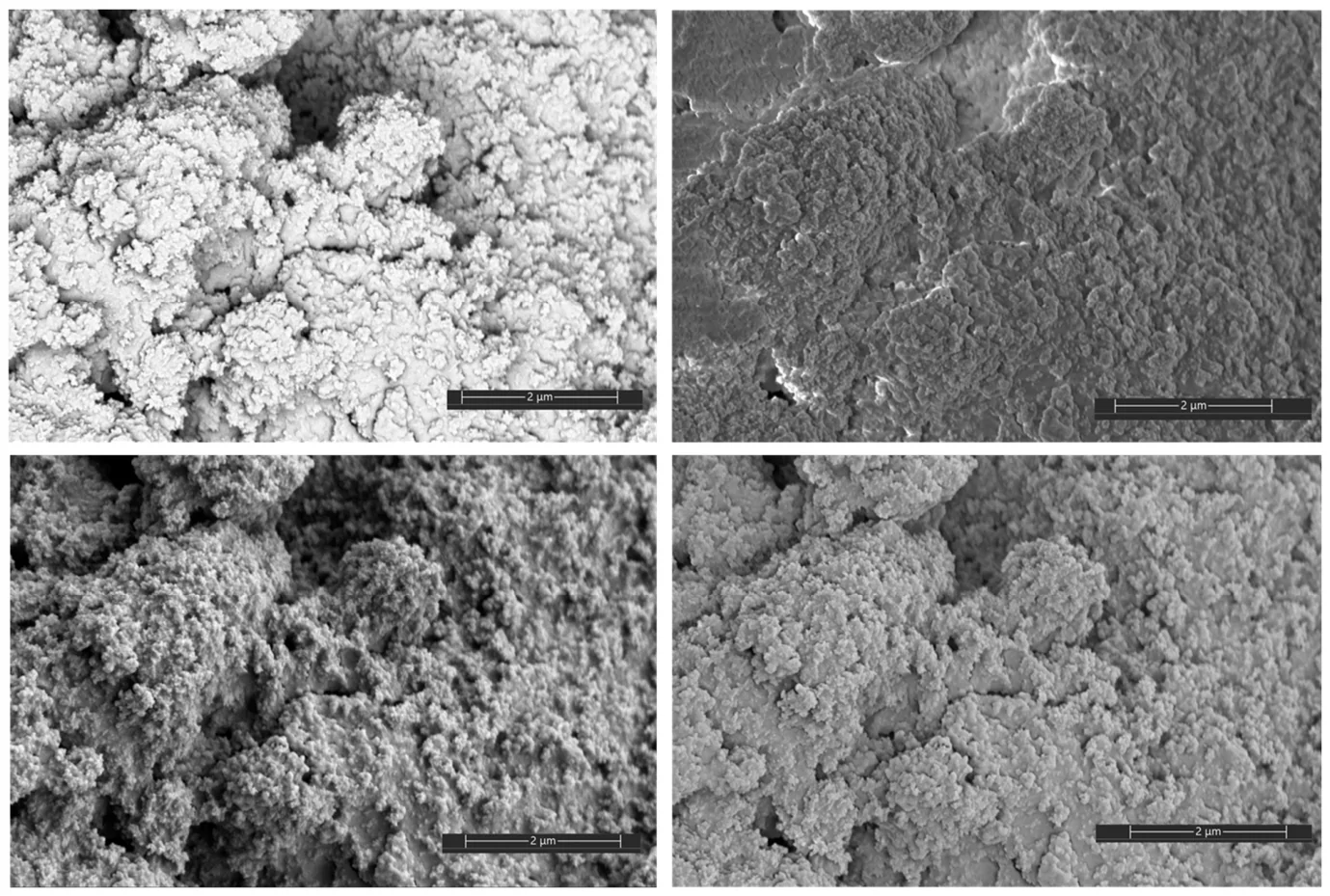
T1, T2, ETD, and Mix SEM images of S1 with ×50,000 magnification.

**Figure 9:**
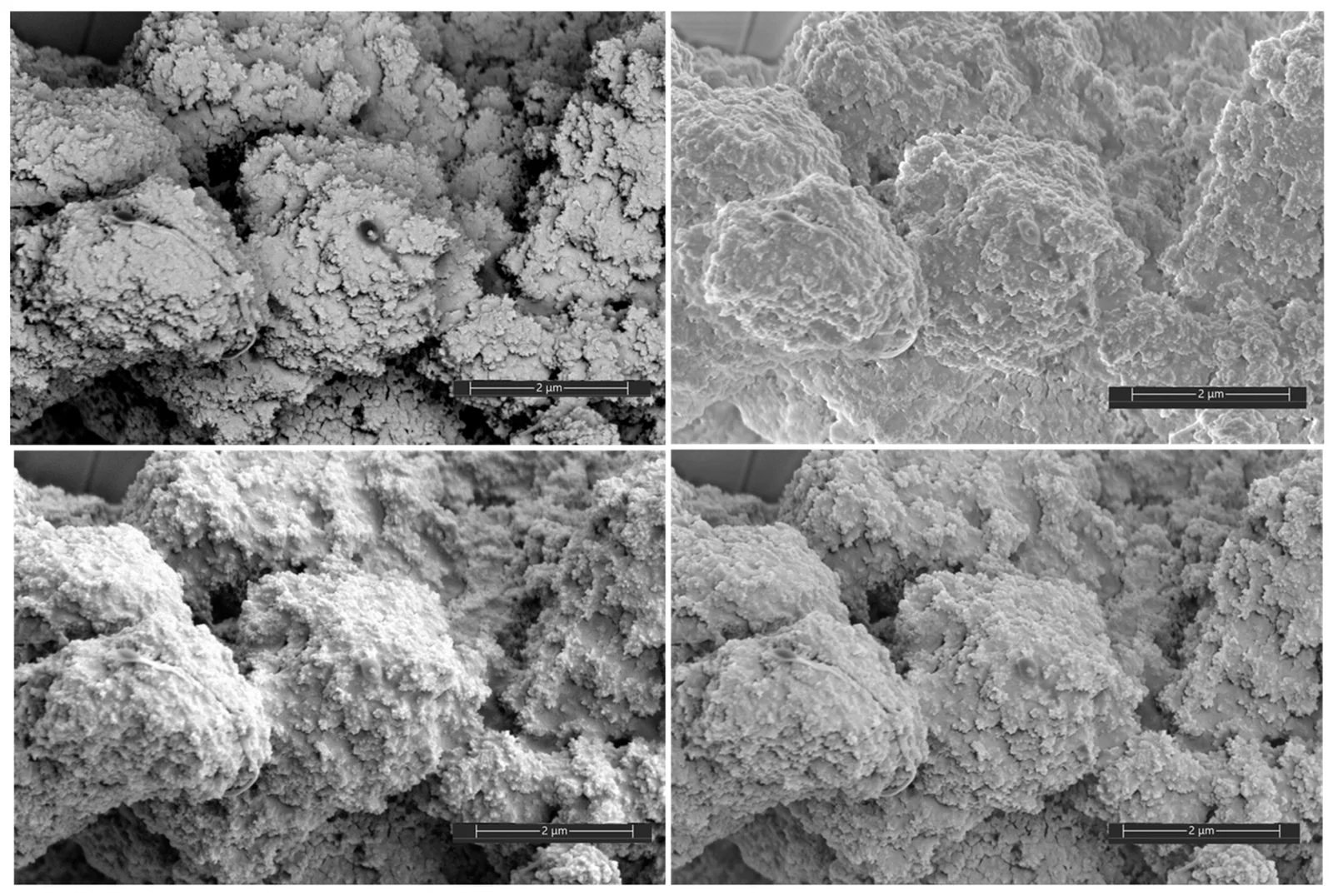
T1, T2, ETD, and Mix SEM images of S2 with ×50,000 magnification.

**Figure 10:**
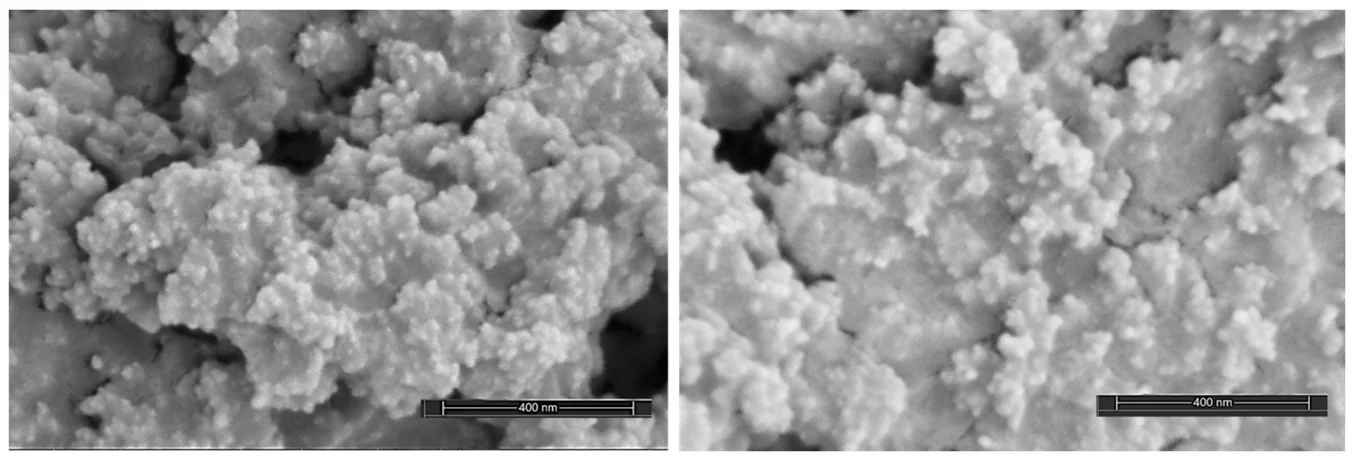
Mix SEM images of S1 with ×300,000 magnification.

**Figure 11:**
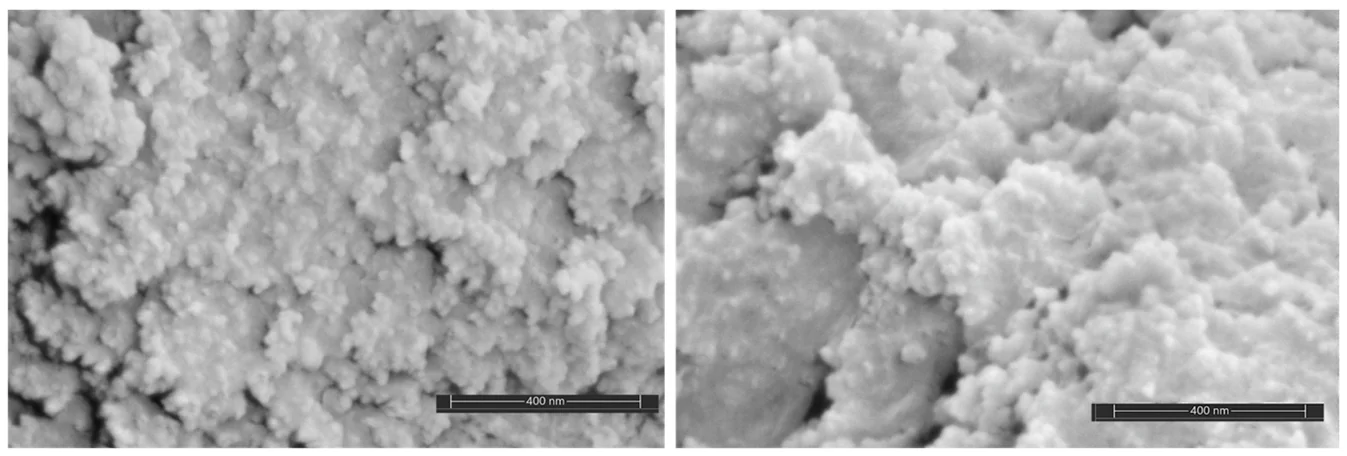
Mix SEM images of S2 with ×300,000 magnification.

Atomic and structural investigations were conducted with a top-down approach for the S1 and S2 compositions. Four different detector pictures were acquired to understand the S1 and S2 structure. The obtained SEM image sequences are shown in Table 2. Therefore, starting at ×1500 magnification, ×50,000 and ×300,000 magnifications were investigated using these four detectors (T1, T2, ETD, Mix) (Table 1), and the resulting images were compared relative to each other. The SEM images at 1500× magnification (Figure 6) of the S1 dried gel revealed a slightly homogeneous structure composed of attached particulate aggregates. The T1 detector emphasizes the surface topography, showing rough agglomerated domains and drying-induced microcracks within the silica-rich matrix. This is quite expected due to the formulation. The T2 image showed additional compositional contrast, and we observed a homogeneous distribution of the particulate structure, with no evidence of phase separation. Also, no aggregates bigger than 50 microns were observed. The ETD detector highlights the irregular surface morphology of the S1 and attached pores, supporting the unexpected formation of a continuous composite network. Finally, the Mix detector combines topographical and compositional information to form the most representative image of the gel structure by simultaneously revealing particle distribution and surface roughness. It can be shown that all four detector modes confirm that the S1 formulation possesses a uniformly distributed, porous silica-based composite structure suitable for dental gel applications. SEM images at 1500× magnification (Figure 7) of the S2 essential oil-containing gel reveal a compact, homogeneous composite morphology that is very similar to that of S1, which is also composed of particulate aggregates. Similarly, the T1 detector emphasizes the relatively rough surface topography and connected agglomerates, indicating improved particle cohesion after essential oil incorporation. The T2 image showed a uniform distribution of both phases. Therefore, we can confirm that the addition of essential oils does not alter the formulation’s crystalline structure. The ETD detector also highlights the porous, cauliflower-like structure characteristic of silica-based gels. In contrast, the Mix detector simultaneously unveils surface roughness and particle distribution, providing the clearest representation of the composite structure. Essentially, the comparison of the four detector modes confirms that the essential oil-containing formulation possesses a slightly more homogeneous, porous microstructure. Still, at this ×1500 magnification, neither S1 nor S2 formulations appear to differ significantly. In the second SEM image comparison, a magnification of ×50,000 was used to compare the two samples. When examined (Figure 8), 50,000× SEM images of the S1 silica-based gel material reveal the nanoscale organization of the composite matrix with excellent structural continuity. The T1 detector in this image shows densely packed spherical particles with a cauliflower-like appearance. Somehow, the T2 detector shows local variations in particle density while confirming the absence of large particle domains or phase separation. The ETD image confirms that the uniformly distributed nanoparticle clusters and interconnected pores dominate the surface. Finally, the Mix detector reveals the homogeneous distribution of silica particles and hierarchical surface roughness. Consequently, the four detectors demonstrate that the S1 gel has a well-integrated nanostructured composite network. Particles in this gel are uniformly dispersed, and surface roughness is evident with structural stability. The 50,000× SEM images (Figure 9) of the S2 essential oil-containing silica-based gel reveal an organized hierarchical structure composed of densely packed nanoparticle aggregates. The T1 detector unveiled similar cauliflower-like structures with fine particles uniformly covering the surface. Compared with S1, the essential oils promote stronger particle agglomeration. In fact, the T2 detector revealed small variations confirming the absence of large particles. The ETD image shows attached nanoparticle clusters and a porous structure, which is typical of silica-based composite gels. Finally, the Mix detector combines topographical and compositional information, providing the most representative visualization of the composite architecture by simultaneously revealing the homogeneous nanoparticle distribution and surface roughness. It can be shown that the four detector modes consistently indicate that incorporating essential oils preserves the particle-oriented morphology while producing a more compact, uniformly distributed, and mechanically integrated composite network, supporting the successful formation of a stable multifunctional gel. For further comparison, only SEM images of the Mix detector for the S1 (Figure 10) and S2 (Figure 11) gels, obtained at 300,000× magnification, were examined.

When examined, S1 images reveal that the surface consists of densely packed spherical silica nanoparticles that are interconnected to form cauliflower-like aggregates with a continuous hierarchical architecture. The nanoparticles, which are about 50 nm in diameter, are uniformly distributed throughout the observed region, with no evidence of large isolated particles, phase separation, or crystalline domains, indicating excellent homogeneity of the silica-rich composite matrix. Uniformity is excellent, as seen in the images.

The high particle density and interconnected nanostructure suggest that the drying process preserved the integrity of the gel network while producing a stable structure.

The Mix detector investigation revealed no particle irregularities, confirming a strong interaction in S1. This regularity provides a high specific surface area, which is useful for the immobilization of bioactive compounds. Also, S2 images acquired at 300,000× magnification reveal a highly compact, homogeneous nanostructured surface with visible particles. The nanoparticles are uniformly distributed across the observed area with no evidence of large crystalline domains, particle segregation, or structural discontinuities, indicating excellent compatibility between the inorganic silica-based matrix, as also observed in S1. Only a few small interparticle voids are observed. The majority of the surface structure seemed densely oriented, suggesting that the essential oil-containing formulation of S2 has good structural integrity. The homogeneous nanoparticle distribution and compact architecture indicate effective incorporation of the bioactive constituents without disrupting the silica network. Since the morphology of S2 looks improved from the SEM images, it is safe to conclude that the S2 formulation is highly suitable for general multifunctional dental gel applications because it provides stability and uniform distribution of active ingredients.

**Figure 12:**
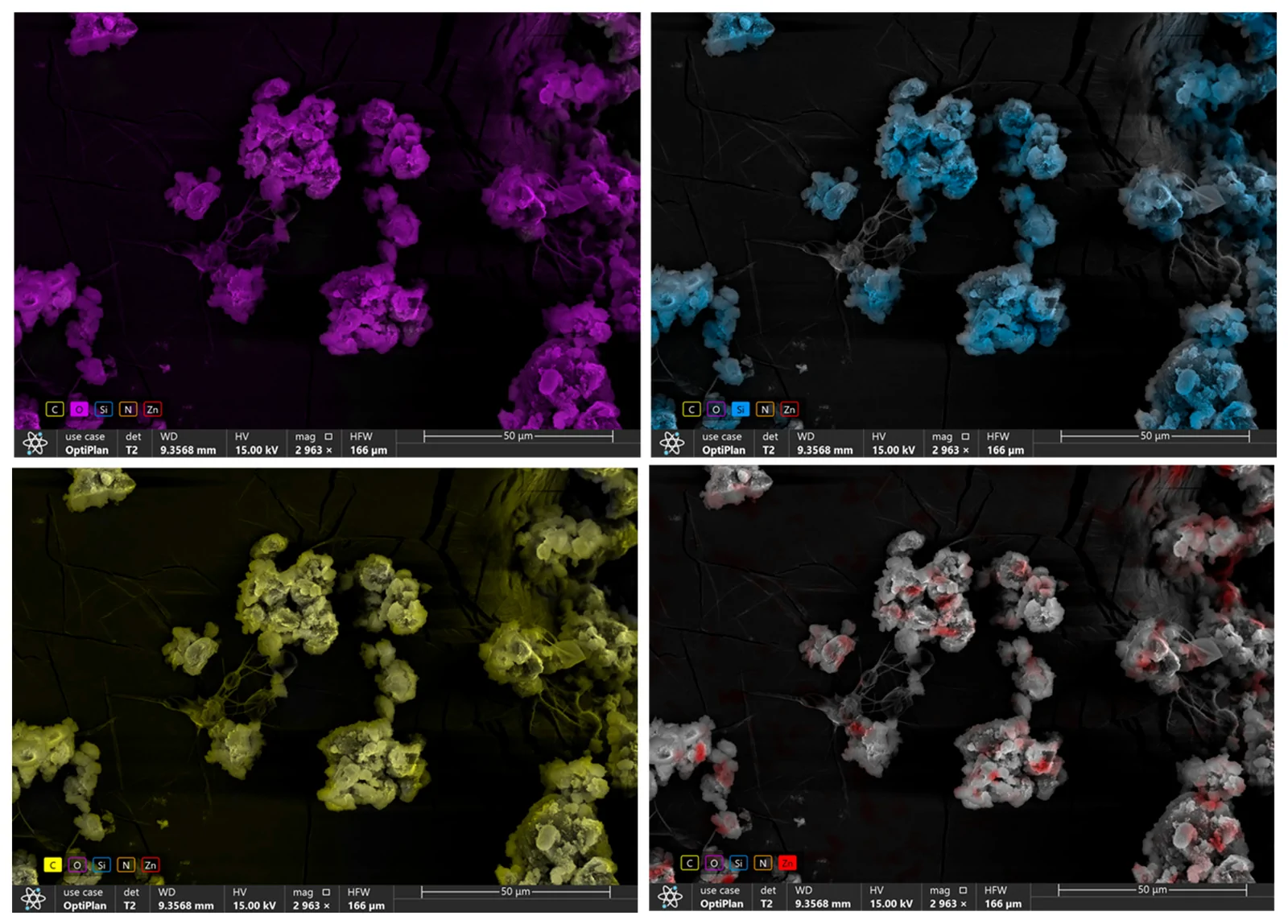
Atomic mapping for S1 gel material for Si, O, C, and Zn.

**Figure 13:**
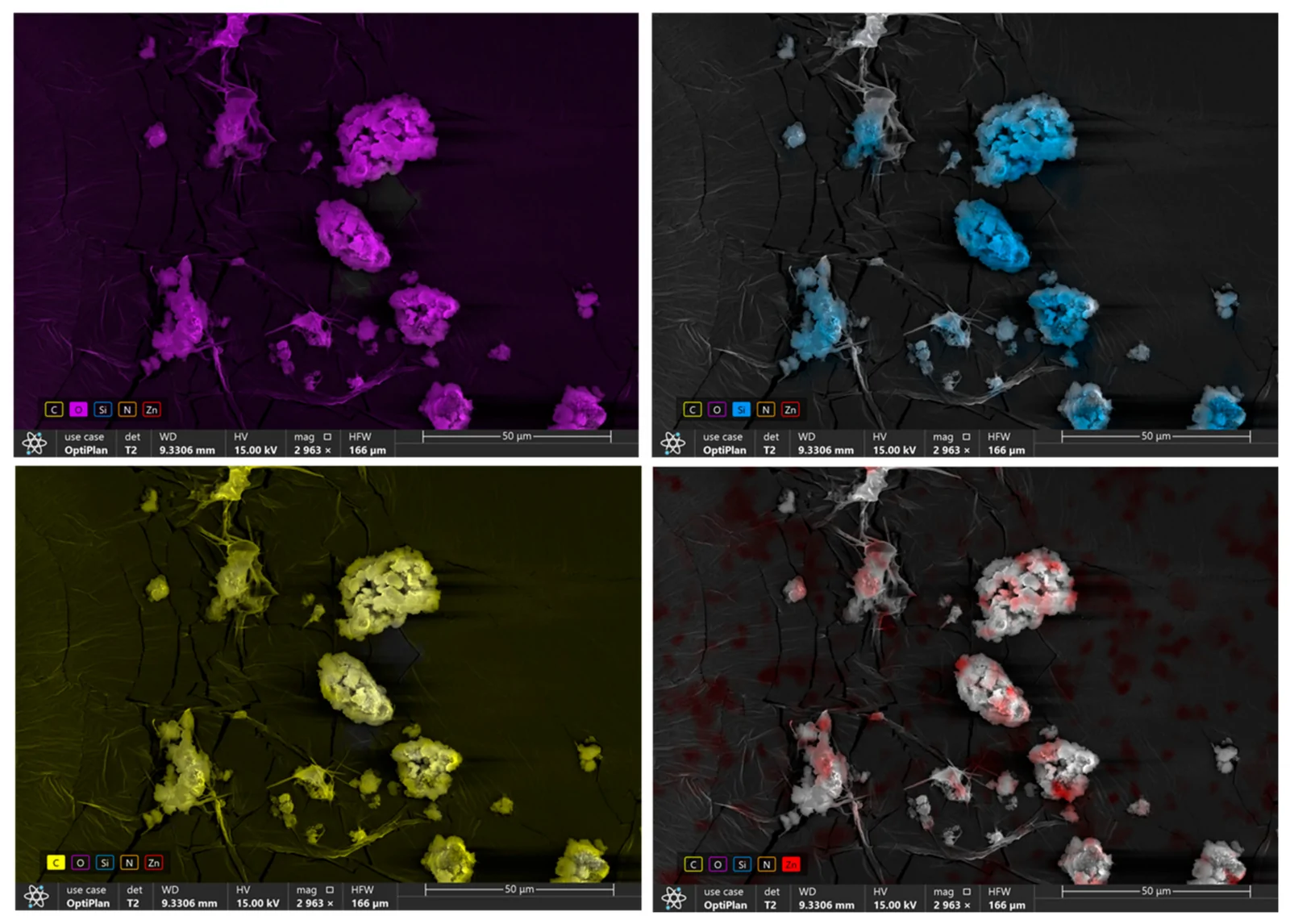
Atomic mapping for S2 gel material for Si, O, C, and Zn.

Comparison of SEM images at different detector modes revealed that both S1 and S2 showed a regular silica-based composite morphology. Attached particles are seen within the porous networks. Still, no phase separation was observed, indicating homogeneous dispersion of the inorganic particles throughout the gel matrix. Also, the elemental mapping results show that both S1 (Figure 12) and S2 (Figure 13) exhibit a homogeneous elemental distribution throughout the silica-based composite gel matrix. In both formulations, C and O are continuously distributed, indicating the presence of organic gel components and oxygen-rich structural groups. Atomic mapping of Si by EDX also revealed a homogeneous distribution of silica particles. The Zn peak is weak in S1 and S2, consistent with the low ZnO content, but remains detectable. Compared with S1, the S2 essential oil-containing formulation shows a more pronounced and continuous carbon distribution, reflecting the contribution of essential oil-derived organic constituents. Finally, we can clearly reveal that the elemental maps confirm that the addition of the essential oils did not cause elemental segregation. S2 formulation maintains the homogeneous silica-based structure while introducing additional organic functionality.

### 3.4. Surface Properties with FT-IR and XPS

**Figure 14:**
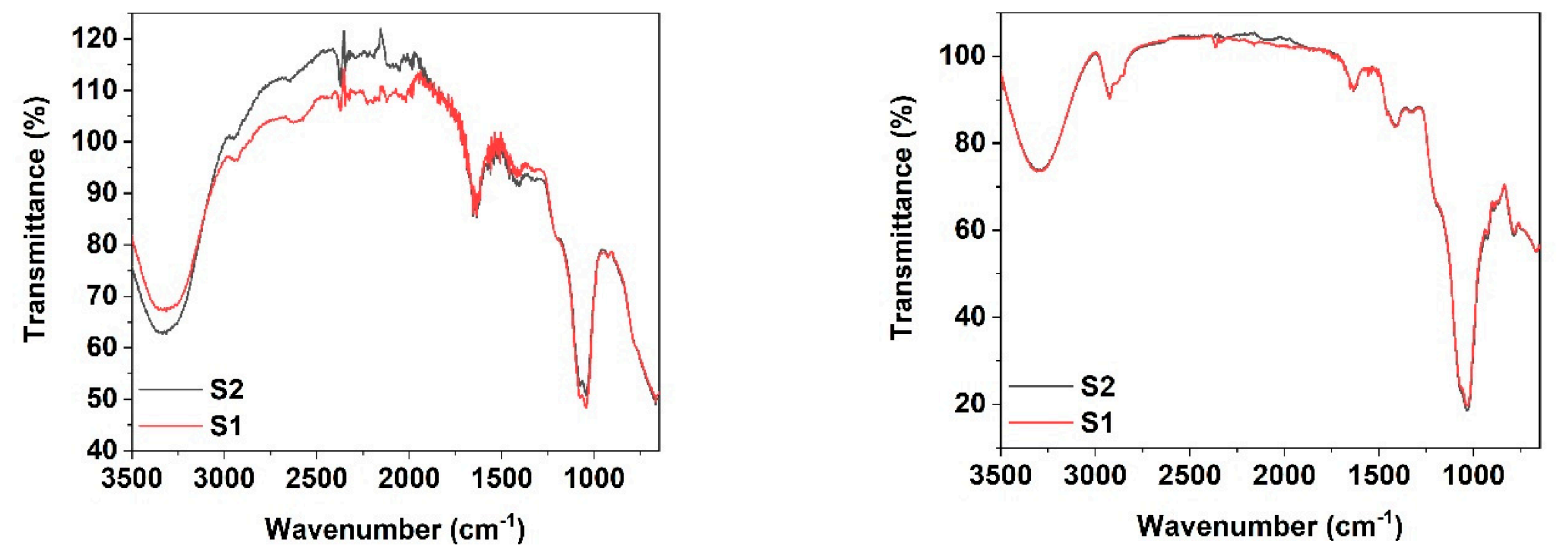
Overlapping FT-IR spectra of gel (**left**) and powder forms (**right**) of the S1 and S2 formulations.

**Figure 15:**
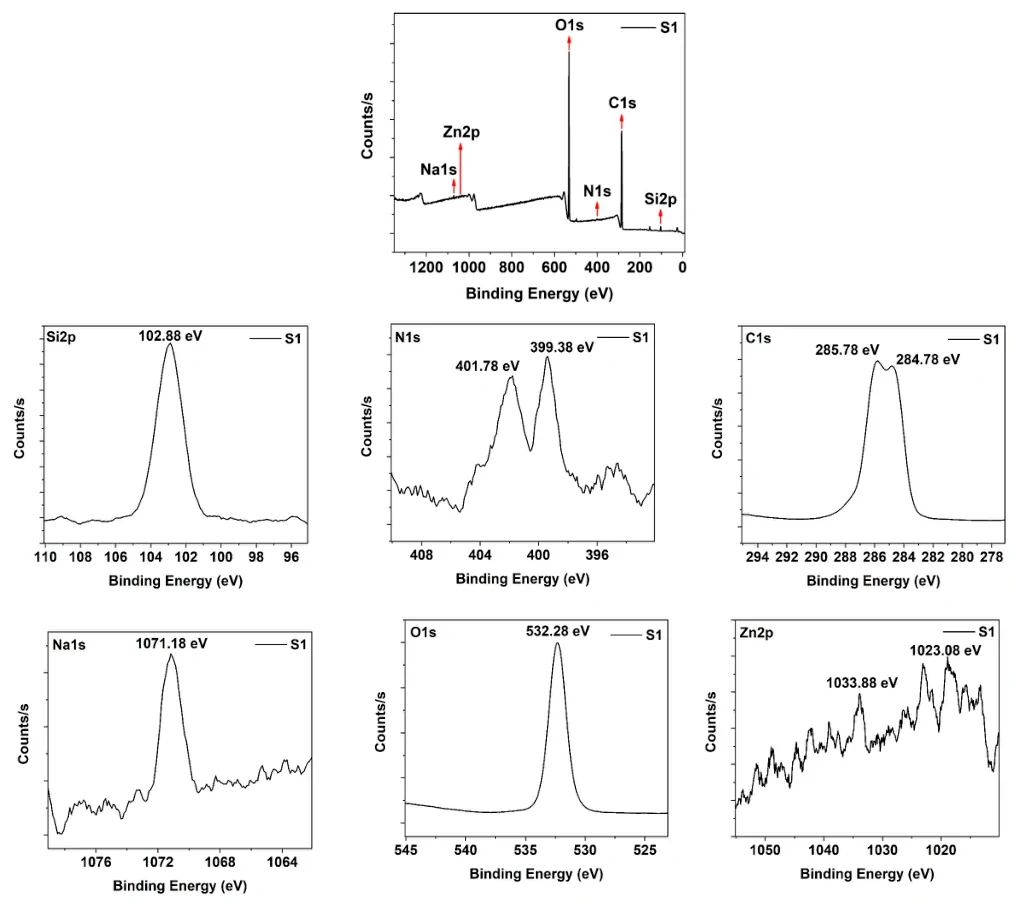
Survey and high-resolution elemental XPS spectra of S1.

**Figure 16:**
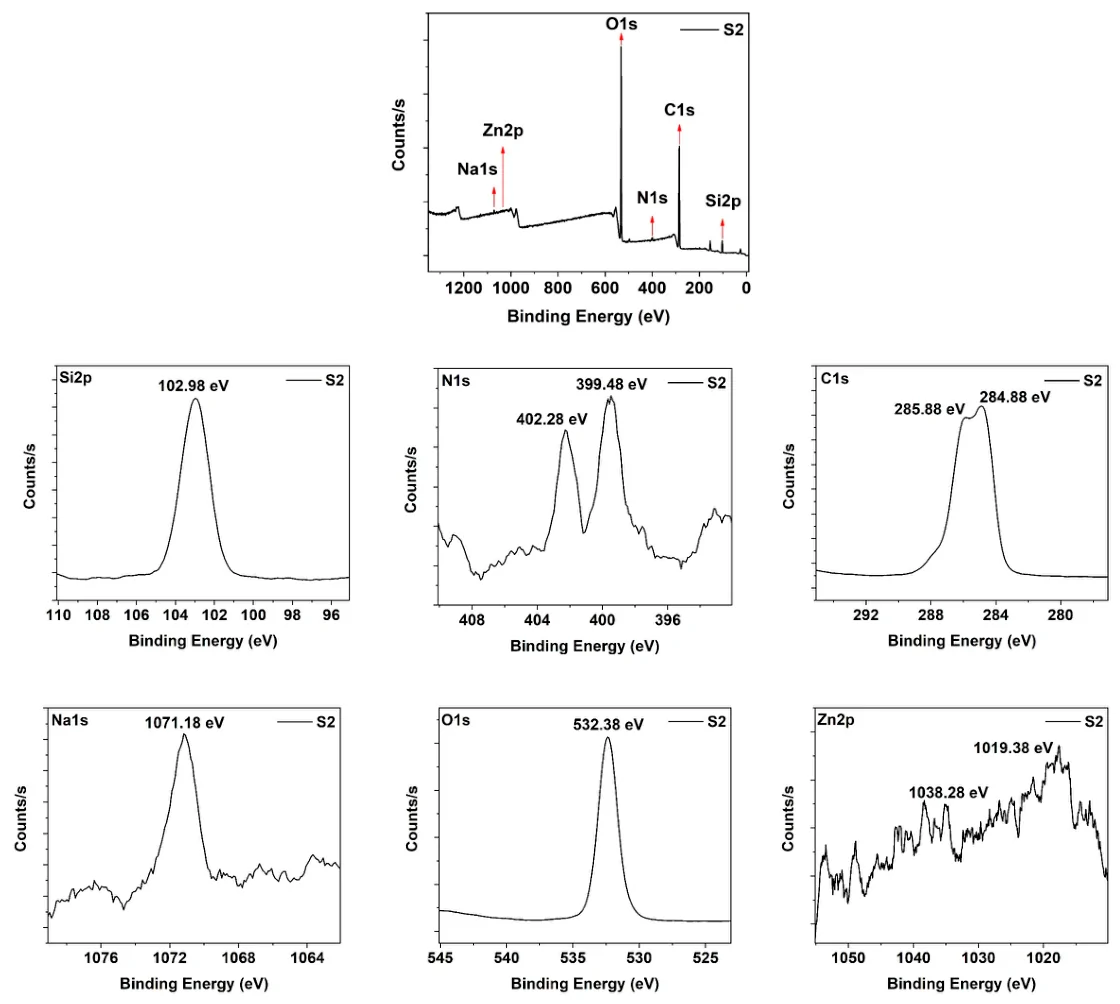
Survey and high-resolution elemental XPS spectra of S2.

**Figure 17:**
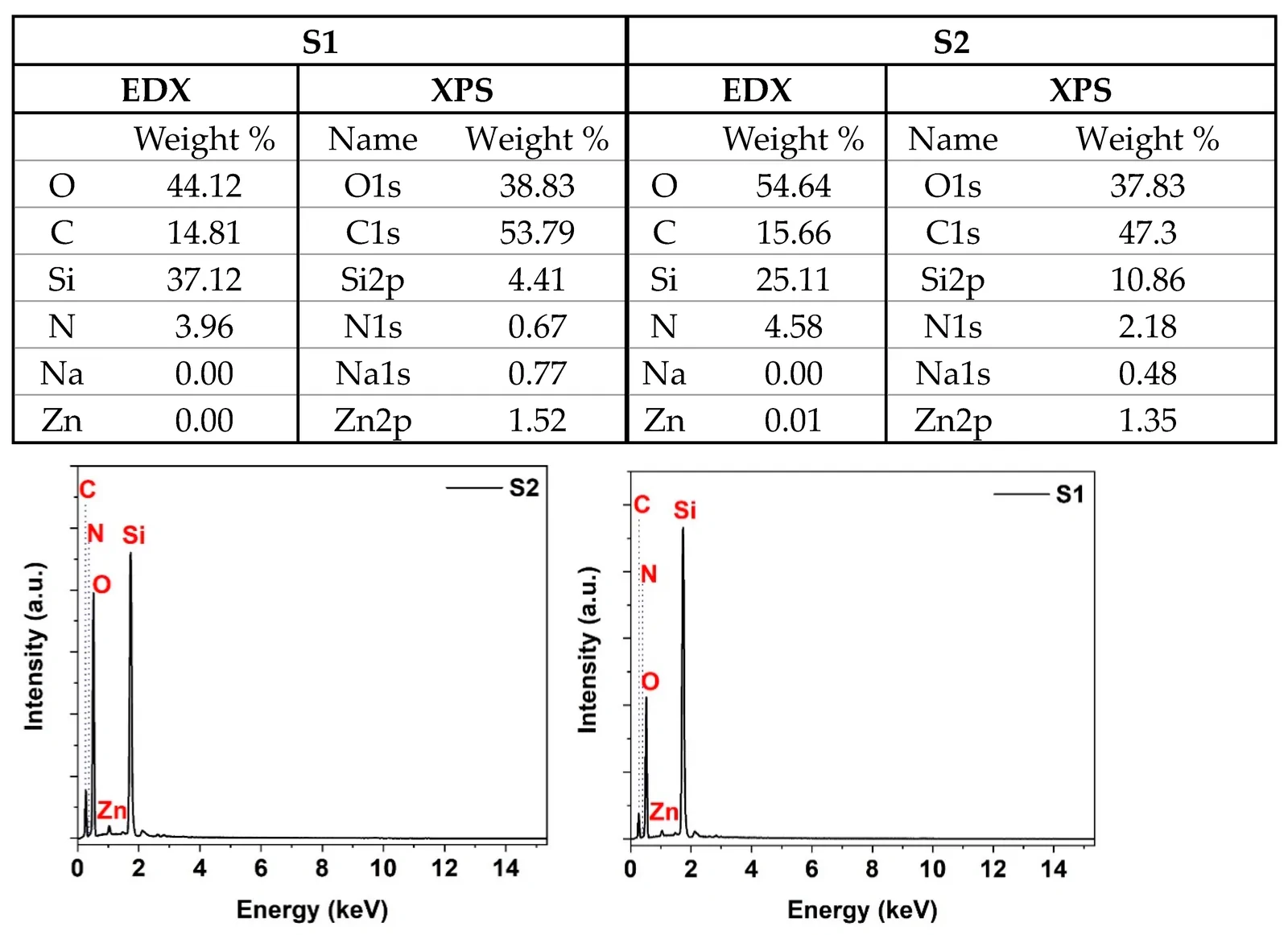
Comparison of the atomic weight content by EDX and XPS investigations and corresponding EDX spectra.

The FTIR spectra (Figure 14) of the control gel (S1) and the essential oil-containing gel (S2) exhibit similar characteristic absorption bands, indicating that incorporation of the essential oils does not alter the fundamental chemical structure of the silica-based composite gel. An observable broad peak between 3600 and 3000 cm^−1^ is known as the stretching vibration of (O–H) groups. These peaks generally originate from absorbed water and OH groups on the silica (e.g., silanol groups). The higher intensity and broader profile of this peak in S2 suggest enhanced hydrogen-bonding interactions resulting from the oxygen-containing constituents of the incorporated essential oils. Weak absorption bands in the 2950–2850 cm^−1^ region generally mean asymmetric and symmetric stretching vibrations of aliphatic C–H groups, while the band around 1630 cm^−1^ is attributed to H–O–H bending vibrations together with contributions from C=C stretching of aromatic and unsaturated phytochemicals present in the essential oils. The increased spectral intensity of S2 in the 1700–1400 cm^−1^ region further supports the successful incorporation of oxygenated organic compounds, including phenolic and terpene-based constituents [40,41,42]. S1 and S2 show an intense absorption peak centered at 1050–1100 cm^−1^, which corresponds to the asymmetric stretching vibration of Si–O–Si bonds. This is possibly due to the silica network remaining the dominant structural framework of the composite gel. The similarity in the position of this band demonstrates that essential oil incorporation does not disrupt the siloxane backbone. In fact, the peaks indicate that surface silanol groups are interacting with organic components. Consequently, observed silica absorption peaks, together with modifications detected in the hydroxyl and organic functional group regions, such as increased C-H and double-bond regions, confirm the addition of essential oils to the gel. During these interactions, the chemical integrity of the silica-based composite formulation remains durable. The FTIR graph of the dried S1 and essential oil-containing S2 gels showed nearly identical absorption peaks, meaning that the drying procedure effectively removed excess moisture, and no change in the chemical structure can be observed. In fact, a smaller peak intensity of the broad O–H stretching band (3600–3000 cm^−1^) confirms successful dehydration. Also, the characteristic Si–O–Si asymmetric stretching band at approximately 1050–1100 cm^−1^ remains clear. Minor variations observed in the 1700–1400 cm^−1^ region are attributed to the organic functional groups of the incorporated essential oils, suggesting weak intermolecular interactions rather than the formation of new chemical bonds. The XPS survey and high-resolution elemental spectra revealed noticeable differences in the surface composition of the two silica-based gel formulations [43,44]. For S1 (Figure 15), the characteristic binding energies were seen at 285.30 eV (C 1s), 532.31 eV (O 1s), 102.93 eV (Si 2p), 399.41 eV (N 1s), 1071.17 eV (Na 1s), and 1018.48 eV (Zn 2p). Also, detected atomic concentrations were 50.71 at.% C, 44.04 at.% O, 3.97 at.% Si, 0.83 at.% N, 0.38 at.% Na, and 0.07 at.% Zn. However, the essential oil-containing S2 (Figure 16) revealed binding energies of 285.25 eV (C 1s), 532.37 eV (O 1s), 102.98 eV (Si 2p), 399.54 eV (N 1s), 1071.16 eV (Na 1s), and 1018.14 eV (Zn 2p), with corresponding concentrations of 45.07 at.% C, 44.44 at.% O, 9.01 at.% Si, 0.98 at.% N, 0.41 at.% Na, and 0.08 at.% Zn. From these investigations, almost the same binding energies of the Si 2p peak and O 1s peaks showed that the incorporation of essential oils did not cause differentiation in the chemical state of the silica framework. Still, the absence of additional peaks indicates that no new contaminant phases are present. Quantitative comparison of elemental weights indicates a possible redistribution of the surface chemistry of S2. The carbon content decreased from 50.71 at.% (S1) to 45.07 at.% (S2)**,** whereas the silicon concentration increased markedly from 3.97 at.% to 9.01 at.%**,** meaning an approximately 2.3-fold increase in surface-exposed silica. Another comparison shows that the C/Si atomic ratio decreased from 12.77 for S1 to 5.00 for S2, suggesting greater exposure of the inorganic silica network at the outermost surface. Similar trends were observed in the weight percentages, where silicon increased from 6.05 wt.% (S1) to 13.31 wt.% (S2)**,** while carbon decreased from 52.64 wt.% to 45.14 wt.%**.** However, the oxygen concentration appears similar (44.04–44.44 at.%), indicating that the oxygen-containing silica structure was preserved. The Zn concentration also remained nearly constant (0.07–0.08 at.%), demonstrating that the low ZnO loading maintained a stable chemical state throughout the synthesis. Figure 17 compares the EDX and XPS atomic ratios for the elemental entities with the EDX spectra.

The same effect was also observed in EDX measurements. XPS results showed that the addition of essential oil does not alter the intrinsic chemical states of SiO_2_ or ZnO. They were affected in the elemental distribution at the surface through intermolecular interactions, which may have resulted in a chemically stable hybrid gel. Also, results clearly revealed that the inorganic framework and the successful integration of bioactive organic constituents formed a new stable gel system. Although logically incorporating essential oil into the S1 formulation should increase the surface carbon content, we concluded that, since the method focuses only on the surface region, the results can be accepted.

### 3.5. Cytotoxicity and Antibacterial Characterization

**Figure 18:**
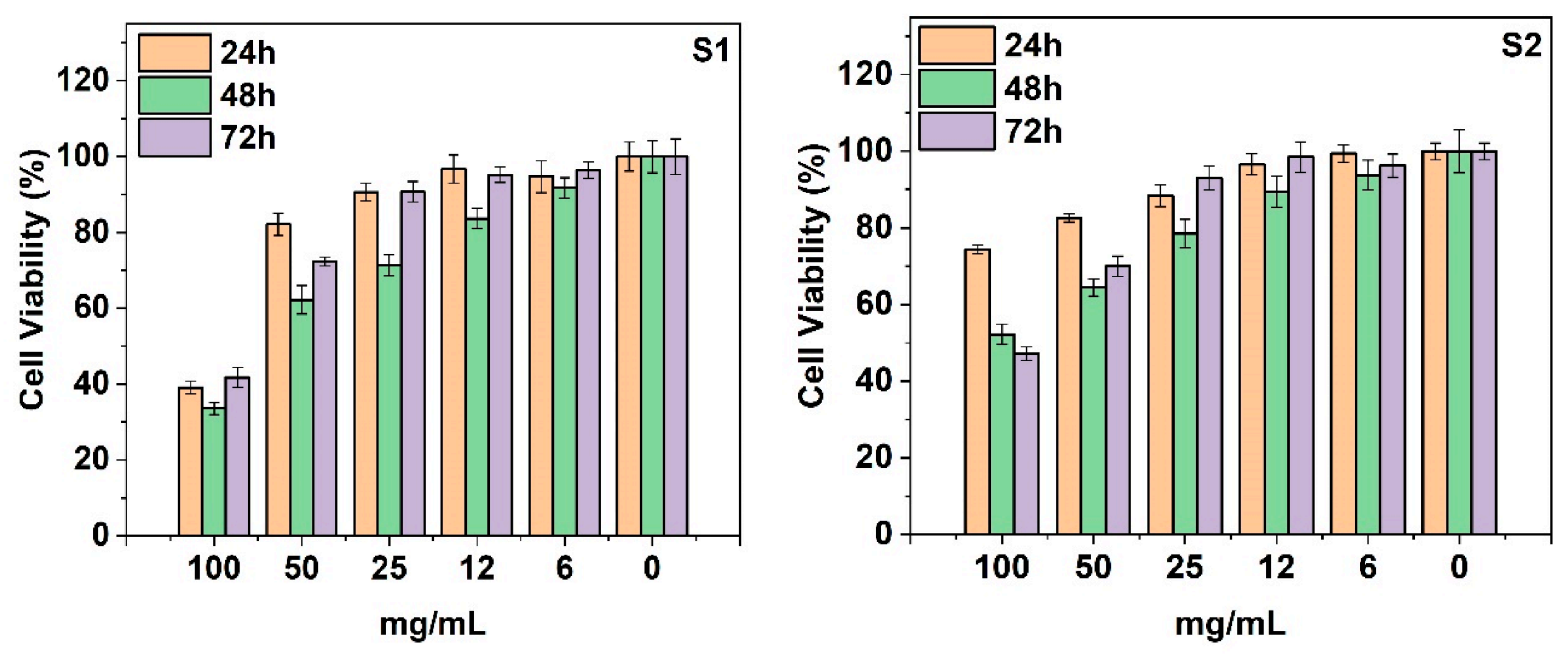
L 929-Cytotoxicity investigations of the S1 and S2 formulations.

The MTT results (Figure 18) demonstrated a clear concentration-dependent cytotoxic response for both S1 and S2 gel formulations [45,46,47]. For the concentration of 100 mg/mL, cytotoxicity was below the commonly accepted threshold, with values of 39.10%, 33.63%, and 41.75% for S1 and 74.25%, 52.20%, and 47.21% for S2 after 24, 48, and 72 h, respectively.

However, at lower concentrations, particularly 25, 12, and 6 mg/mL, both formulations showed markedly improved cytocompatibility. For S1, viability was 90.63–96.66% at 24 h and 90.73–96.33% at 72 h, while S2 showed 88.41–99.43% at 24 h and 93.01–98.44% at 72 h within the same concentration range. These results indicate that both silica-based gel systems are cytocompatible at optimized concentrations, whereas excessive formulation concentration may induce cellular stress. Compared with S1, the essential oil-containing formulation S2 generally exhibited comparable or improved biocompatibility, especially at biologically relevant lower concentrations. The high viability values observed for S2 at 25 mg/mL (93.01%), 12 mg/mL (98.44%), and 6 mg/mL (96.26%) after 72 h confirm that incorporation of essential oils did not introduce significant cytotoxicity into the gel matrix.

## 4. Conclusions

In this current study, we created a gel that is mainly filled with silica and has some ZnO, with the special addition of natural oils. XRD analysis showed that the gel did not show crystallinity due to the silica structure, but also the addition of natural oils did not affect the overall structure of the produced gel formulation. The SEM observations showed that the obtained oil-containing gel S2 looks almost the same when compared with the non-oil-containing structure of S1. ZnO detection can only be seen in EDX and XPS characterization, which highlights the low amount of ZnO. XPS results mostly indicate that the carbon amount varies after the addition of natural oils. Also, FTIR spectroscopy showed that the S1 and S2 gels still have the same peaks as silica and ZnO and the natural oils did not change the general representation. Similarly, UV–Vis and photoluminescence analyses demonstrated that the optical properties were preserved after essential oil incorporation, while thermal analysis showed that both formulations followed similar multistep degradation pathways, with only quantitative differences arising from the additional volatile organic fraction introduced by the essential oils. MTT assays revealed that the essential oil-containing silica composite formulation showed cytocompatibility with L929 fibroblasts. The combination of structural robustness, favorable biocompatibility, and non-cytotoxicity highlights the synergistic effect between the silica/ZnO matrix and bioactive essential oils. Collectively, these complementary characterization results showed that the incorporation of multiple natural essential oils modifies the surface chemistry and thermal behavior of the silica-rich gel without compromising its structural stability. This comprehensive physicochemical evaluation provides new insights into the design of multifunctional silica-based gel formulations for advanced dental applications and establishes a useful platform for future investigations.

## Figures and Tables

**Table 1:** Thermal loss amounts for different temperature windows.

Sample	0–100 °C (%)	100–255 °C (%)	255–360 °C (%)	360–900 °C (%)	Residue at 900 °C (%)
S1	32.64	17.93	22.03	4.47	22.93
S2	36.84	15.18	25.28	4.8	17.90
Dried S1	5.33	14.94	32.23	6.74	40.76
Dried S2	5.88	17.79	37.63	7.45	31.25

**Table 2:** Obtained and presented SEM image orientations and explanation of detector types.

Detector	Signal	Main Characteristics
T1	Secondary electrons (SEs)	High surface sensitivity, fine surface morphology
T2	(SE) + compositional contribution	Greater depth and material contrast than T1
ETD (Everhart–Thornley Detector)	Secondary electrons	Classical topographic SEM
Mixed Detector	Combination of SE+ Backscattering Electrons	Simultaneous morphology and compositional contrast

## Data Availability

The original contributions presented in this study are included in the article. Further inquiries can be directed to the corresponding author.
